# A novel polysaccharide-based bioflocculant produced by *Bacillus subtilis* 35A and its application in the treatment of dye decolorization, heavy metal ion adsorption and meat product wastewater

**DOI:** 10.3389/fmicb.2024.1457909

**Published:** 2024-08-22

**Authors:** Jinping Dai, Xingxiu Zhao, Shengdong Mu, Qinghuan Yang, Changqing Zhao, Zhifeng Zhao

**Affiliations:** ^1^College of Bioengineering, Sichuan University of Science and Engineering, Yibin, China; ^2^College of Biomass Science and Engineering, Sichuan University, Chengdu, China

**Keywords:** *Bacillus subtilis* 35A, bioflocculant, dyes decolorization, heavy metals adsorption, organic wastewater treatment

## Abstract

Optimizing the fermentation process of microorganisms with exceptional bioflocculant-producing capabilities is crucial for the production of bioflocculants. The application of bioflocculants to various pollutants highlights their significant advantages in water treatment. Therefore, the culture conditions of *Bacillus subtilis* 35A with exceptional bioflocculant-producing capabilities were optimized. The bioflocculant (MBF) was obtained by alcohol percipitation from the fermentation supernatant, and its physicochemical properties were analyzed to explore its application in the treatment of dyes, heavy metal ions, and organic wastewater. The results indicate that, using cyclodextrin and yeast extract as carbon and nitrogen sources, after 48 h of fermentation at the initial pH, the bioflocculant (MBF-35A) yielded 10.47 g/L with a flocculation rate of 96.57% for kaolin suspension. The chemical analysis demonstrated that MBF-35A is mainly composed of polysaccharide (81.74%) and protein (16.42%). FITR and XPS analysis indicated that MBF-35A mainly contains major elements such as carbon, nitrogen, and oxygen, with functional groups (-OH, C-O, C-H, and C-O-C) that are beneficial for flocculation. MBF-35A exhibited a dye decolorization efficiency exceeding 95% and removed 41.05 and 48.93% of Cr^6+^ and Cu^2+^ ions, respectively. In meat wastewater treatment, the effective removal rates of ammonia nitrogen (26.87%), COD (51.16%), total nitrogen (37.76%), and total phosphorus (55.81%) highlight its potential in organic waste treatment. In brief, not only does MBF-35A exhibit efficient production and excellent flocculation performance as a bioflocculant, but it also shows significant biological and environmental benefits in dye, heavy metal ions, and organic wastewater treatment.

## 1 Introduction

Inorganic and synthetic organic coagulants are widely utilized in industrial applications due to their excellent coagulation performance, stability, and cost-effectiveness. However, their poor biodegradability raises concerns regarding environmental safety and public health (Ayangbenro et al., 2019; Fan et al., 2019). For instance, aluminum salts, a major component of inorganic coagulants, are associated with potential risks of Alzheimer’s disease, while acrylamide monomers exhibit high carcinogenicity and neurotoxicity in humans (Hassimi et al., 2020). Therefore, the urgent development of safe and biodegradable coagulants is imperative.

Compared to conventional coagulants, microbial flocculants (MBF) have attracted significant scientific and technological attention in wastewater treatment due to their safety, non-toxicity, high flocculation efficiency, ease of biodegradation, non-hazardous degradation intermediates, and broad adaptability to pH changes (Li et al., 2023). MBF is a specialized metabolite produced by microorganisms or their secretions, exhibiting flocculation activity and serving as a novel green and environmentally friendly flocculant (Xia et al., 2022). It’s particularly noteworthy that attention is directed toward polysaccharide-based bioflocculants due to their unique structure, high thermal stability, excellent rheological properties, and selectivity (Huang et al., 2019). They possess significant potential in treating various industrial wastewaters, including heavy metal wastewater (Ayangbenro et al., 2019), dye wastewater (Pu et al., 2020; Wang et al., 2020), drinking water treatment (Giri et al., 2015), and microalgae recovery (Sivasankar et al., 2020), among others.

However, the high cultivation cost and complex fermentation process pose major bottlenecks to the large-scale application of MBF. Therefore, it is essential to screen for novel bioflocculant-producing strains with high activity and stability, as well as strains capable of utilizing inexpensive and non-toxic nutrient sources. Simultaneously, optimizing fermentation conditions is crucial for enhancing bioflocculant yield (Xia et al., 2022). Most of the current studies have optimized the culture conditions of microorganisms (carbon and nitrogen sources, temperature, pH, culture time, etc.). The commonly used carbon sources in the production of bioflocculants include glucose, starch, sucrose, etc., and the commonly used nitrogen sources include urea, yeast powder, beef extract, peptone, etc. Microorganisms have a specific affinity for substrates. The optimal carbon and nitrogen sources of different microorganisms are often different, and the structure of the flocculant produced is also different (Zhao et al., 2023). Zhao et al. (2017) proved that glucose was the best carbon source for the production of flocculant by *Bacillus subtilis* CZ1003 through single-factor experiments. In the experiment of Rasulov et al. (2017), D-mannose was more suitable as a carbon source for the production of flocculant by *rhizobiumSZ4S7S14* than glucose. *S. pavanii* GXUN74707 uses glucose as the carbon source and urea as the nitrogen source (Qin et al., 2024). The temperature in the culture process mainly affects the flocculant production by affecting the activity of related enzymes. Similarly, the effect of the initial pH of the medium on flocculant production cannot be ignored. The pH mainly affects flocculation activity by affecting the absorption of nutrients by microorganisms and the activity of cell enzymes. Based on a variety of factors that affect the production of bioflocculants, it can be seen that optimizing the optimal culture conditions is an indispensable step in the production of bioflocculants.

In the previous experiment, it was found that *Bacillus subtilis* 35A had good flocculation activity on kaolin suspension. *Bacillus subtilis* 35A came from the preserved strain in our laboratory, which was previously isolated from sludge and stored in 20% glycerol at −80°C. In this study, optimization of cultivation conditions for *Bacillus subtilis* 35A, a high-efficiency producer of bioflocculants, was initially conducted to enhance flocculation activity and bioflocculant yield. The physicochemical and characterization properties of the bioflocculant produced in this study (MBF-35A) were investigated, elucidating its flocculation mechanism. Subsequently, MBF-35A was applied to treat dye decolorization, adsorption of heavy metal ions, and removal of organic compounds in the wastewater of meat products. The superior pollutant removal capacity of MBF-35A in practical applications highlights its biotechnological potential.

## 2 Materials and methods

### 2.1 Strain producing bioflocculant

The strain was initially activated at 30°C and 150 rpm in a nutrient broth medium containing 10 g/L peptone, 9 g/L beef extract, and 5 g/L NaCl. Afterward, it was inoculated in a bioflocculant-producing medium (Zhao et al., 2017), comprised of 20 g/L glucose, 9 g/L beef extract, 0.75 g/L KCl, and 0.5 g/L NaCl for enrichment.

### 2.2 Determination of flocculation activity

Kaolin tests were carried out to determine the flocculation activity. A 25-mL glass tube containing 10 mL of kaolin clay suspension (4 g/L), 0.05 mL of CaCl_2_ solution (1% w/v), and 0.2 mL of fermentation broth was vortexed for 20 s, and then left to stand for 30 min (Xia et al., 2022). The absorbance of the supernatant was measured at 550 nm. A control was prepared using the same method, but the fermentation broth was replaced by a fresh medium. The flocculation activity was determined according to the following Equation 1:


(1) $$\mathrm{Flocculation}\;\mathrm{Activity}\;\left(\%\right)=\frac{A-B}{A}\times 100\%$$

Where A and B are the optical densities of the control and the sample at 550 nm, respectively.

### 2.3 Optimization of cultivation conditions for *Bacillus subtilis* 35A

To obtain the optimum flocculating activity for *Bacillus subtilis* 35A, eight factors including carbon sources (glucose, fructose, sucrose, maltose, lactose, corn starch, cyclodextrin, dextrin, soluble starch, sodium acetate, and mannitol), nitrogen sources (peptone, beef extract, yeast extract, urea, (NH_4_)_2_SO_4_, and NH_4_Cl, glycine, and diammonium citrate), phosphate level (1.25 g/L K_2_HPO_4_ + 0.5 g/L KH_2_PO_4_, 2.5 g/L K_2_HPO_4_ + 1 g/L KH_2_PO_4_, 5 g/L K_2_HPO_4_ + 2 g/L KH_2_PO_4_ and 10 g/L K_2_HPO_4_ + 4 g/L KH_2_PO_4_), metal ions level (single 0.2 g/L MgSO_4_, 0.1 g/L NaCl, 0.5 g/L KCl and the combination of 0.2 g/L MgSO_4_,0.08 g/L NaCl, 0.46 g/L KCl), initial pH of the medium (natural, 4, 5, 6, 7, 8, 9 and 10), incubation temperature (25°C, 30°C, 35°C and 40°C), inoculation size (0.5, 1, 2, 4and 6% (v/v)) and time (12 h,24 h,48 h,60 h,72 h,84 h and 96 h) were investigated. 1% (v/v) of seed culture was inoculated into a 250-mL Erlenmeyer flask containing 100 mL of fresh liquid medium and incubated aerobically at 30°C and 150 rpm on a rotary shaker. Samples were taken at 48 h. Each flask experiment was carried out in triplicates.

### 2.4 Production and extraction of MBF-35A

The culture system was amplified to 1 L in a 2 L Erlenmeyer flask under the optimum conditions for the enrichment of fermentation broth. After 48 h of incubation, the fermentation broth was collected and centrifuged at 10,000 g, 4°C for 15 min to remove bacterial cells. The supernatant was mixed with 95% ethanol and left overnight at 4°C for biopolymer precipitation (Wang et al., 2018). The precipitate was obtained by centrifugation at 10,000 g at 4°C for 15 min, washed once with ethanol, and finally lyophilized to obtain the bioflocculant MBF-35A, whose yield was determined by weighing. The flocculation efficiency of MBF-35A on kaolin was measured according to method 2.2, with 5 mg of MBF-35A replacing 0.2 mL of fermentation broth.

### 2.5 Physical and chemical analysis of MBF-35A

The chemical composition of MBF-35A was analyzed. The total sugar content of MBF-35A was measured by the phenol-sulfuric acid method, with glucose as the standard solution. The total protein content of MBF-35A was determined by the BCA Protein Assay Kit with bovine serum albumin as the standard solution (Jingqiu, 2021). The elemental composition of MBF-35A was determined using X-ray photoelectron spectroscopy (XPS, Shimadzu/Kratos AXIS SUPRA+, Japan), scanning was carried out over a wide binding energy range (0–1,100 eV). The C1 signal peak (284.6 eV) was chosen as an internal reference to calibrate the position of other peaks. The functional group of MBF-35A was analyzed by Fourier transform infrared (FTIR) spectroscopy (NEXUS 670, Nicolet, America). Dried sample powder taken in KBr pellet was conducted at a resolution of 2 cm^−1^, between 4,000 and 400 cm^−1^. The surface morphology of MBF-35A were observed by scanning electron microscopy (SEM).

### 2.6 Application of MBF-35A in treating different pollutants

#### 2.6.1 Dyes decolorization by MBF-35A

Methylene blue and toluol blue were used as the target dyes in the present study. Different dosages of MBF-35A (2–70 mg) were added to a 50-mL dye solution (10, 20, 50, and 100 mg/L), and the mixture was agitated at 160 rpm for 24 h. After centrifugation at 10,000 g for 10 min, the supernatant was measured with a UV–Vis spectrophotometer (UV2600, Shimadzu, Japan) at the maximum absorption wavelength of each dye. The color removal rate was calculated as follows Equation 2:


(2) $$\mathrm{Dye}\;\mathrm{removal}\;\mathrm{rate}\;\left(\%\right)=\frac{c_{o}-c_{e}}{c_{o}}\times 100\%$$

Where C_0_ and C_e_ were the initial and final concentrations of the dye.

solution, respectively.

#### 2.6.2 Heavy metal adsorption by MBF-35A

2–50 mg of MBF-35A powder was added into 50 mL of different heavy metal ion solutions (50 mg/L Cr_2_O_7_^2^and 50 mg/L Cu^2+^), respectively. These solutions were agitated in a shaker at 160 rpm for 24 h and then centrifuged at 10,000 g for 10 min to remove the insoluble flocs. The residual amounts of metal ions in the supernatant were measured with an atomic absorption spectrophotometer (SP-3500AA, Shanghai Spectrum Instrument Co., Ltd.). The heavy metal removal rate was calculated as follows Equation 3:


(3) $$\mathrm{Heavy}\;\mathrm{metal}\;\mathrm{removal}\;\mathrm{rate}\;\left(\%\right)=\frac{M_{o}-M_{e}}{M_{o}}\times 100\%$$

Where M_0_ and M_e_ were the initial and final concentrations of the metal solution, respectively.

#### 2.6.3 Treatment of wastewater from meat products by MBF-35A

Different doses of MBF-35A powder (2–20 mg) were individually added to 100 mL of wastewater from a meat processing plant. The mixtures were stirred at 80 rpm for 30 min, followed by filtration of flocs through a 0.22 μm water-soluble membrane filter. Water quality parameters, including ammonia nitrogen, chemical oxygen demand (COD), total nitrogen, and total phosphorus of the wastewater before and after treatment, were measured using a multiparameter water quality analyzer (GL-200, GLKRUI, China). The removal efficiency was calculated as follows Equation 4:


(4) $$\mathrm{Removal}\;\mathrm{efficiency}\;\left(\%\right)=\frac{H_{o}-H_{e}}{H_{o}}\times 100\%$$

Where H_0_ and H_e_ were the initial and final values of the wastewater solution, respectively.

The water quality parameters of the tested wastewater samples: COD (450 mg/L), ammonia nitrogen (57.11 mg/L), total nitrogen (65.33 mg/L), total phosphorus (2.12 mg/L), and pH (7.3–8.5).

### 2.7 Analytical methods

Optical density measured at 600 nm (OD_600_) was utilized to describe bacterial growth. The pH was measured by a digital pH meter. Zeta potential was determined by the Zetasizer Nano zs90. The micromorphology of bacterial strains and kaolin particles was observed using SEM (Nova Nano, SEM 230).

### 2.8 Statistical analysis

All data were obtained from triplicate experiments and subjected to one-way analysis of variance (ANOVA) using SPSS 17.0 statistical software (α = 0.05). A *p*-value of less than 0.05 was considered statistically significant.

## 3 Results and discussion

### 3.1 Physicochemical and characterization of *Bacillus subtilis* 35A

**Figure 1:**
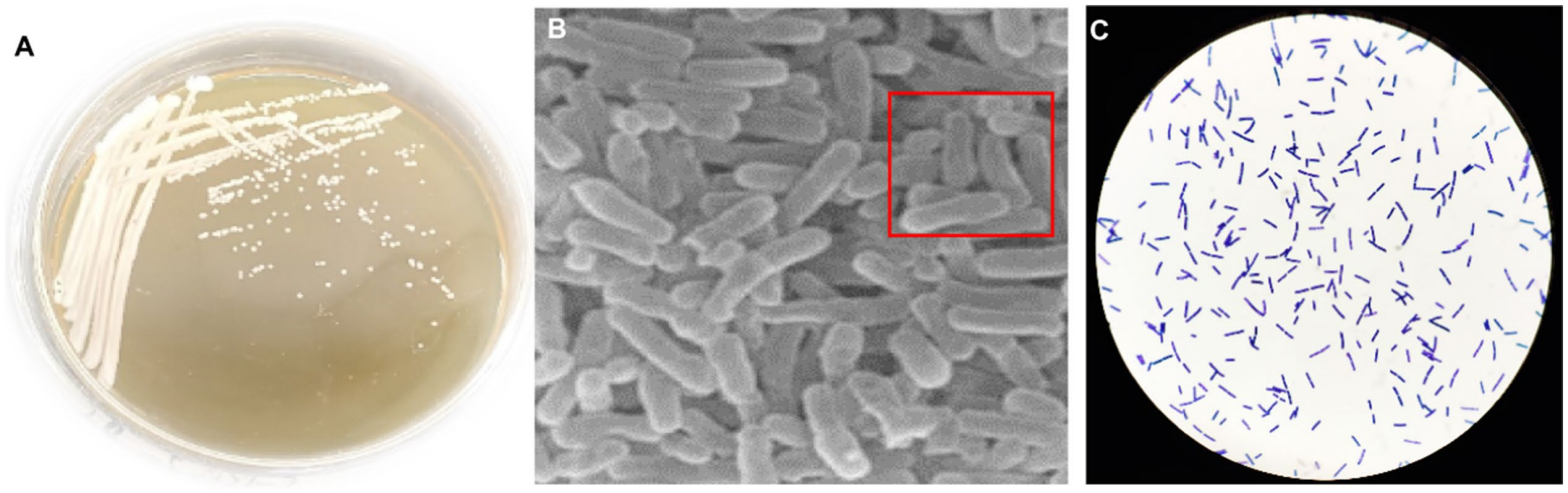
Colony morphology **(A)**, cell micromorphology **(B)** and gram stain **(C)** of *B. subtilis* 35A.

Colonies of *Bacillus subtilis* 35A on nutrient agar solid medium appeared as irregular, opaque, and slightly rough-edged pale-yellow forms (Figure 1A). Microscopic examination revealed that the cells of this strain were typical short rods (Figure 1B), and Gram staining showed positive results (Figure 1C).

### 3.2 Effect of carbon and nitrogen sources on MBF production from *Bacillus subtilis* 35A

**Figure 2:**
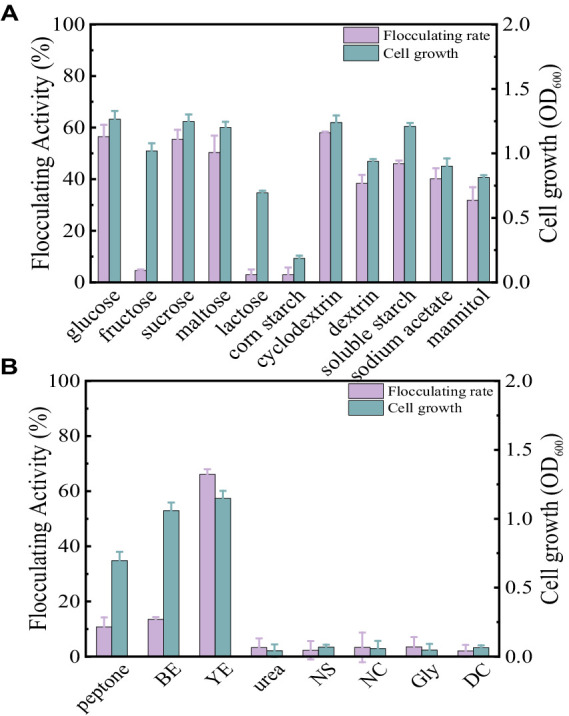
Comparison of flocculating activity and bacterial growth of *B. subtilis* 35A in different carbon sources **(A)** and **(B)** nitrogen sources (BE: beef extract, YE: yeast extract, NS: (NH_4_)_2_SO_4_, NC: NH_4_Cl, Gly: glycine, DC: diammonium citrate).

Carbon sources play a key role in enhancing bioflocculant synthesis by microorganisms. In this section, the concentration of carbon sources was fixed at 10 g/L. As displayed in Figure 2A, glucose, sucrose, maltose, cyclodextrin, dextrin, soluble starch, sodium acetate, and mannitol all appeared to be favorable for *Bacillus subtilis* 35A to produce bioflocculant. They achieved high flocculating activity and cell growth, particularly with glucose, sucrose, and cyclodextrin. Fructose and lactose supported the bacterial growth, while almost no bioflocculant was excreted. Additionally, corn flour as the carbon source induced both poor cell growth and low flocculating activity. Considering the high flocculating activity and its potential as a cheap substrate in practical applications, cyclodextrin was selected as an appropriate carbon source in this study.

Generally, organic nitrogen sources contain amino acids and vitamins, which help to improve bioflocculant production. At a fixed cyclodextrin concentration of 10 g/L and a nitrogen source concentration of 5 g/L, the effect of different nitrogen sources on flocculation activity is shown in Figure 2B. Only yeast extract powder resulted in high bioflocculant production by *Bacillus subtilis* 35A, and the flocculating activity reached 66.11% at 48 h. Peptone and beef extract can maintain cell growth, but the flocculation activity is not high. Urea, (NH_4_)_2_SO_4_, NH_4_Cl, glycine, and diammonium citrate caused both low flocculation activity and poor cell growth. Thus, yeast extract was the best nitrogen source for *Bacillus subtilis* 35A. Due to The diversity of carbon and nitrogen source types and the differences in the microorganisms studied (Table 1), the carbon and nitrogen sources applicable to the production of bioflocculants vary greatly among different microorganisms (Ntsangani, 2017; Qin et al., 2024).

**Table 1:** Examples of bioflocculant producing microorganisms in literature.

Strains	Based medium	MBF yield	Flocculating activity	References
*Pseudomonas koreensis* and *Pantoea* sp (Ayangbenro et al., 2019)	2 g/L glucose, 5 g/L peptone, 5 g/L yeast extract, 5 g/L K_2_HPO_4_, 2 g/L KH_2_PO_4_, 1 g/LNH_4_Cl,0.5 g/L MgSO_4_, 0.2%CaCO_3_ (30°C, pH 7.2)	2.98 g/L,3.26 g/L	71.3,51.7%	Polysaccharides, proteins
*Klebsiella variicola* B16 (Xia et al., 2018)	10 g/L glucose, 1 g/L (NH_4_)_2_SO_4_, 5 g/L K_2_HPO_4_, 2 g/L KH_2_PO_4_, 0.2 g/L MgSO_4_, 0.1 g/L NaCl (30°C, pH7.0)	6.96 g/L	Not reported	Polysaccharides, proteins
*Alteromonas* sp. CGMCC 10612 (Chen et al., 2017)	30 g/L glucose, 1.5 g/L wheat flour, 1 g/L KH_2_PO_4_, 5 g/L K_2_HPO_4_, 30–40 g/L sea salt (25°C, pH7.5)	11.18 g/L	2575.4 U/mL	Polysaccharides (69.61%), protein (21.56%)
*Bacillus nitratireducens* (B4) (Abbas et al., 2020)	-	-	90%	Polysaccharides, proteins
*Klebsiella oxytoca* GS-4-08 (Fan et al., 2019)	50 mM, KH2PO4 100 mM, MgSO4 1 mM, CaCl2 0.1 mM, glucose 8 g/L, SL-6 trace element solution (0.1% v/v), 1 g/L acetonitrile (ACN)	Not reported	90%	Polysaccharides (46.3%), protein (20.6%)
*Bacillus subtilis* ZHX3 (Xia et al., 2022)	10 g/L starch, 5 g/L yeast extract, 5 g/L NaCl (30°C, natural pH6.74)	3.14 g/L	95.5%	Polysaccharides (77.2%), protein (14.8%)
*S. pavanii* GXUN74707 (Qin et al., 2024)	10 g/L of glucose, 0.5 g/L of yeast extract, 0.5 g/L of urea, 5 g/L of K_2_HPO_4_, 2 g/L KH_2_PO_4_, 0.1 g/L NaCl, and 2 g/L MgSO4 · 7H2O, pH 7.0–7.5.	-	99%	Carbohydrates (79.70%), protein (14.38%)
*Bacillus subtilis* 35A	10 g/L cyclodextrin, 9 g/L yeast extract, 0.2 g/L MgSO4 + 0.5 g/L KCl (35°C, natural pH6.81)	10.47 g/L	96.57%	This study

-: Not reported.

### 3.3 Effect of carbon and nitrogen source concentrations, phosphate salts, metal ions, initial pH, inoculum size, temperature and time on MBF- 35A production

**Figure 3:**
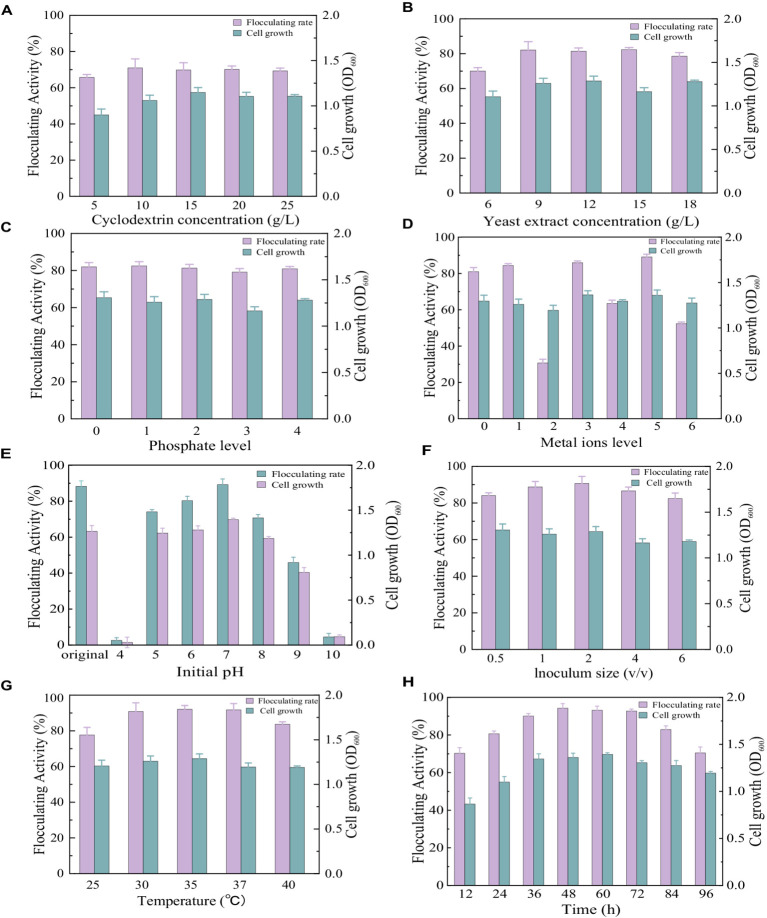
Comparison of flocculating activity and bacterial growth of *Bacillus subtilis* 35A in different parameters: **(A)** Cyclodextrin concentration, **(B)** Yeast extract concentration, **(C)** phosphate level (1: 1.25 g/L K_2_HPO_4_ + 0.5 g/L KH_2_PO_4_, 2: 2.5 g/L K_2_HPO_4_ + 1 g/L KH_2_PO_4_, 3: 5 g/L K_2_HPO_4_ + 2 g/L KH_2_PO_4_, 4: 10 g/L K_2_HPO_4_ + 4 g/L KH_2_PO_4_), **(D)** Metal ions level (1: 0.2 g/LMgSO_4_, 2: 0.1 g/LNaCl, 3: 0.5 g/L KCl,4:0.2 g/LMgSO_4_ and 0.1 g/LNaCl,5:0.2 g/LMgSO_4_ and 0.5 g/L KCl, 6:0.1 g/LNaCl and 0.5 g/L KCl), **(E)** Initial pH, **(F)** Inoculum size, **(G)** Temperature, **(H)** Time.

As shown in Figure 3A, similar cell growth appeared in the medium with different cyclodextrin concentrations. The flocculating activity reached 65.69, 70.97, 69.83, 70.17, and 69.29% at cyclodextrin concentrations of 5 g/L, 10 g/L, 15 g/L, 20 g/L, and 25 g/L, respectively. There was no significant difference in flocculating activity when the cyclodextrin concentrations ranged from 10 g/L to 25 g/L. To reduce the cost of cultivation, the cyclodextrin concentrations were set at 10 g/L. When cyclodextrin was fixed at 10 g/L, similar cell growth appeared in the medium with different yeast extract concentrations (Figure 3B). The flocculating activity reached 70.03, 82.14, 81.42, 82.31, and 78.60% at yeast extract concentrations of 6 g/L, 9 g/L, 12 g/L, 15 g/L, and 18 g/L, respectively. There was no significant difference in flocculating activity when the yeast extract concentrations ranged from 9 g/L to 15 g/L. To reduce the cost of cultivation, the yeast extract concentration was set at 9 g/L.

As displayed in Figure 3C, phosphate supplementation at all tested levels had no significant effect on the ability of *Bacillus subtilis* 35A to produce bioflocculant. It has been reported that phosphate, as a good buffer, plays a key role in regulating pH during fermentation (Chen et al., 2017). Thus, it has been widely applied in bioflocculant producing mediums. Considering that the addition of phosphate had no significant effect on the flocculating activity and growth of the strain, phosphate was not added in the subsequent culture.

Trace metal ions are required for cellular metabolic activity and enzyme regulation. Both the Na^+^ group and the Na^+^ containing group inhibited bioflocculant production significantly. The addition of Mg^2+^ and K^+^ presented an obvious effect on flocculating activity (Figure 3D). So Mg^2+^ and K^+^ were selected as the trace metal elements in this work. Adding metal ions might be a relatively cheaper way to improve flocculating activity than increasing carbon and nitrogen sources. It has been reported that the addition of Fe^3+^ facilitated starch consumption by strain ZHX3 and improved the stability of flocculating activity between parallel groups (Xia et al., 2022).

The initial pH of the medium will affect the electrification state of cells and redox potential, consequently influencing nutrient assimilation and enzymatic reactions (Zhao et al., 2016). The results of the initial pH effects are depicted in Figure 3E. Flocculating activity remained relatively constant at a range of pH 6.0–8.0, while strong acidic (pH 4.0) and alkaline (pH 10.0) conditions totally inhibited cell growth and MBF-35A production. The maximum flocculating activity (89.19%) was reached at pH 7, which was close to that (88.25%) of the natural pH (6.81). To reduce the extra consumption of acid and alkali, the initial pH was not adjusted for the subsequent experiment.

Inoculum size is a significant factor in bioflocculant production and cell growth. A small inoculum size can prolong the lag phase, whereas a large inoculum size can cause the strain’s niches to overlap excessively, thus inhibiting bioflocculant production (Xia et al., 2022). In this study, 2% (v/v) inoculum size was optimal with the highest flocculating activity of 90.66%, and further increasing it caused a decrease in flocculating activity (Figure 3F). The highest MBF-W7 production by *Bacillus* sp. was obtained with a 5% inoculum size, and its flocculating activity was 87% (Okaiyeto et al., 2016a). It consumed more seed culture. The highest MBF-ZHX3 production by Bacillus sp. was obtained with a 1% inoculum size, and its flocculating activity was 92.4% (Xia et al., 2022).

Culture temperature has a direct effect on microbial enzymatic activity, which further affects bioflocculant production (Li et al., 2023). As displayed in Figure 3J, the flocculation activity reached 90.86, 92.08, and 91.71% at 30°C, 35°C, and 37°C, respectively. 35°C was applied in the subsequent experiments. The similar cell growth of *Bacillus subtilis* 35A indicated its strong survival ability in different temperature conditions, which was favorable for responding to environmental changes. For *Bacillus subtilis* F9, when the temperature ranged from 30°C to 40°C, its flocculation activity increased, but the biomass accumulation decreased correspondingly (Giri et al., 2015).

In the optimum culture medium, the time course assay of MBF-35A production was conducted in an amplified system (1 L), as shown in Figure 3K. After 36 h of cultivation, active cell growth and a dramatic increase in flocculating activity were observed, indicating that the optimized conditions were beginning to take effect and were even more effective in the scale-up medium. The flocculating activity kept increasing and peaked at 94.24% at the 48 h, which was parallel to cell growth, suggesting that MBF-35A was produced by metabolites rather than cell lysis. Thereafter, the flocculating activity decreased slightly until the 96 h, while the bacteria continued growing. It might be attributed to the fact that the soluble starch was completely depleted at 72 h, at this point, the produced bioflocculant could be consumed by the cells as a substitute for food because of inadequate nutrient supply (Xia et al., 2022).

Consequently, the optimized culture conditions were set as 10 g/L cyclodextrin, 9 g/L yeast extract, 0.2 g/L MgSO_4_ + 0.5 g/L KCl, an original pH of about 6.81, 2% (v/v) inoculum size, 35°Cand 48 h.

### 3.4 Extraction of MBF-35A

**Figure 4:**
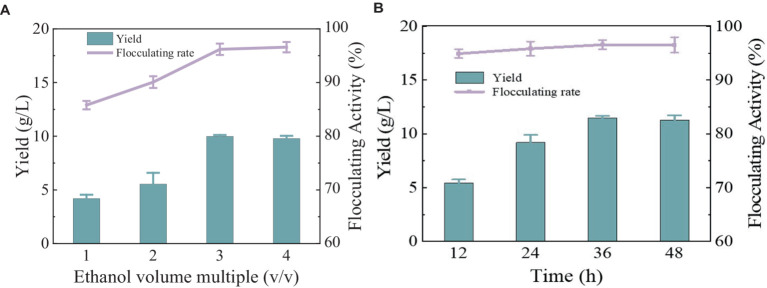
Comparison of ethanol volume **(A)** and alcohol settling time **(B)** on flocculation activity and yield of MBF-35A.

After the fermentation broth was centrifuged to remove the bacteria, anhydrous ethanol with different volume multiples was added for precipitation. The yield and flocculation activity obtained by 3 times and 4 times the volume of anhydrous ethanol were not significantly different and were relatively higher than the other two groups (Figure 4A). Considering the cost, three times anhydrous ethanol was selected for subsequent experiments. The precipitation rate of bioflocculant is also one of the important factors affecting the production cycle. Usually, the longer the time is, the more target products will be obtained. There was no difference in the flocculation activity of the bioflocculant precipitating at different times in Figure 4B, indicating that the time did not affect the flocculation effect. The obvious precipitation phenomenon occurred at 12 h, and the yield did not change significantly after 36 h. A total of 10.47 g/L of MBF-35A was extracted from the fermentation broth. Compared with the reported bioflocculant-producing microorganisms (Table 1), *Bacillus subtilis* 35A possessed relatively high flocculating activity, an average level of MBF yield, simple and low nutritional requirements, and mild culture conditions.

### 3.5 Characterization of MBF-35A

#### 3.5.1 Chemical composition analysis

Chemical analysis revealed that MBF-35A is composed of 81.74% polysaccharides, 16.42% protein, and 0.42% nucleic acids, confirming that MBF-35A is primarily a polysaccharide-like substance. The polysaccharide content is higher than the reported polysaccharide-based bioflocculant (Pu, 2020; Pi, 2021).

#### 3.5.2 FTIR spectrum analysis

**Figure 5:**
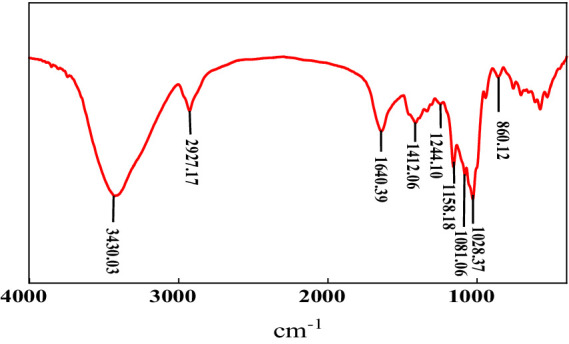
FTIR spectrum of MBF-35A.

The FTIR spectrum of MBF-35A displayed broad stretching peaks (Figure 5). The broad intense peak at 3430.03 cm^−1^ is caused by the stretching vibration of -OH in the sugar ring of polysaccharides. The peak at 2927.17 cm^−1^ is attributed to C-H stretching vibration. The peaks at 1640.39 and 1412.06 cm^−1^ show the presence of C=O stretching vibration and C-H bending vibration, respectively. The peak at 1244.10 cm^−1^ is induced by >P=O stretching vibration. The peaks at 1158.18, 1081.06, and 1028.37 cm^−1^ all indicate the appearance of C-O-C stretching vibration, corresponding to the characteristic peaks of sugar derivatives. The absorption peak at 860.12 cm^−1^ indicates the presence of a α glycosidic bond in the polysaccharide chain (Gong et al., 2008; Liu et al., 2016). In summary, the FTIR spectra provided characteristic functional groups of hydroxyl, carboxyl, and carbonyl, suggesting that the primary components of MBF-35A were polysaccharides, which is consistent with the chemical analysis results.

#### 3.5.3 XPS analysis

**Figure 6:**
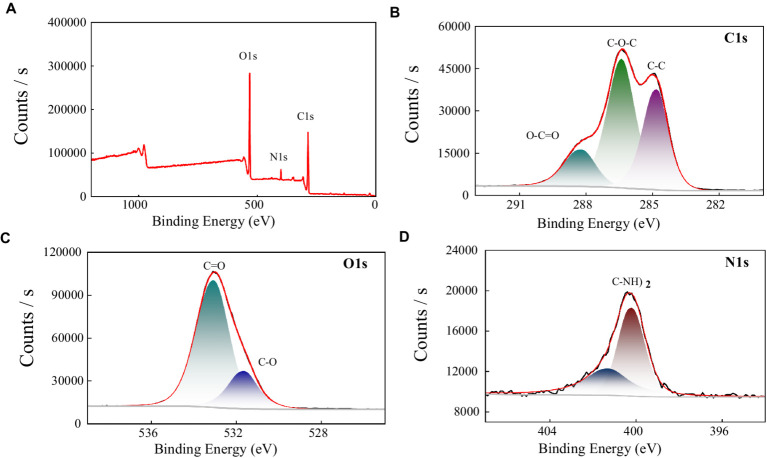
XPS spectra of MBF-35A **(A)** and high resolution 1 s XPS spectra of C, O, and N fromMBF-35A are shown in **(B–D)**, respectively.

In order to further explore the functional groups, and elemental composition of the polysaccharide MBF-35A, we used X-ray photoelectron spectroscopy to analyze MBF-35A in the range of 0–1,100 eV. It can be seen from the full spectrum analysis of Figure 6A that there are three main peaks in the figure, which are C 1 s (286.3 eV), O 1 s (532.7 eV), and N 1 s (400.0 eV), indicating that BM2 mainly contains these three elements. The proportion of different elements was obtained by calculation, C: O: N = 63.02: 32.85: 4.14, and the proportion of elements in the sample was C > O > N. C (47.4%), O (41.9%), and N (5.2%), were detected as the main elements. The low proportion of N elements was consistent with the lower content of protein components in MBF-35A.

To further explore the composition of functional groups on different elements, we performed narrow-spectrum high-resolution scans of C, N, and O in MBF-35A. The narrow spectrum analysis of the C 1 s element (Figure 6B) shows that C 1 s is mainly divided into three peaks, and their positions are 284.8 eV, 286.40 eV, and 288.21 eV, respectively. The binding energy peak at 284.8 eV is attributed to C-C and C-H. The peak of binding energy at 286.40 eV is attributed to C-O and C-N bonds, which may be some amide groups in MBF-35A; the binding energy peak at 288.21 eV is attributed to C = O, which may be the carboxyl, acetal, and hemiacetal structures in MBF-35A (Jingqiu, 2021). The narrow-band high-resolution analysis of O 1 s shows that there are two peaks of O element (Figure 6C), located at the binding energys of 531.62 eV and 533.07 eV, respectively. The peak at 531.62 eV is mainly attributed to the C-O bond, which is mainly carboxyl, carbonyl, or amide in MBF-35A. The second peak (533.07 eV) is mainly attributed to alcohols, hemiacetals, and acetals in MBF-35A (Hu et al., 2020). The narrow spectrum analysis of N1s shows that there are two peaks (Figure 6D) with binding energies of 400.01 eV and 401.28 eV. The first peak at 400.01 eV may be the non-protonated nitrogen element in amines or amino compounds. The second peak is located at 401.28 eV, which often appears in amino acids and amino sugars (Pu, 2020).

### 3.6 Exploration of flocculation mechanism induced by MBF-35A

The surface electrical properties of flocs were tested. The average zeta potential value of the kaolin suspension was negatively charged (−36.2 ± 0.23 mV). After adding MBF-35A alone, the zeta potential of the mixture became more negative (−42.7 ± 3.16 mV), indicating that MBF-35A chains were also negatively charged. In the presence of Ca^2+^ alone, the zeta potential increased obviously to −22.6 ± 0.21 mV, while the addition of bioflocculant induced a slight increase in zeta potential (24.3 ± 0.52 mV). Therefore, charge neutralization proceeds only by the addition of Ca^2+^ in the flocculation system, and it is not the main flocculation mechanism of MBF-35A.

**Figure 7:**
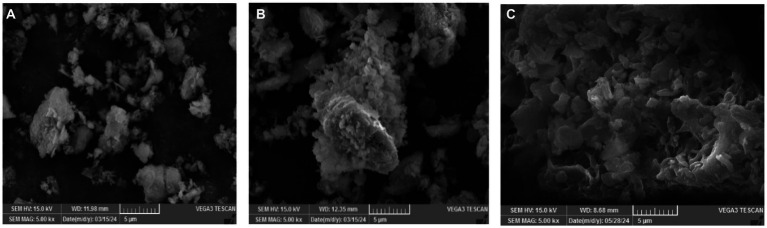
SEM images of kaolin particles **(A)**, sediment induced by Ca^2+^ **(B)** and flocs induced by Ca^2+^ and MBF-35A **(C)**.

The SEM image showed that kaolin particles were scattered before the flocculation process (Figure 7A). The addition of Ca^2+^ induced the simple superimposition of kaolin particles under the effect of charge neutralization (Figure 7B). After adding Ca^2+^ and MBF-35A, a flocculation reaction occurred; kaolin particles were tightly aggregated and settled in the form of larger flocs (Figure 7C). These flocs had a morphology distinct from that of the precipitated kaolin particles induced by charge neutralization. These SEM images provided additional evidence that the specific interactions among MBF-35A, Ca^2+^, and kaolin particles are mainly mediated by adsorption and bridging mechanisms (Aljuboori et al., 2015; Okaiyeto et al., 2016b). These SEM images provided additional evidence that the specific interactions among MBF-35A, Ca^2+^, and kaolin particles are mainly mediated by adsorption and bridging mechanisms.

### 3.7 Decolorization of dyes by MBF-35A

**Figure 8:**
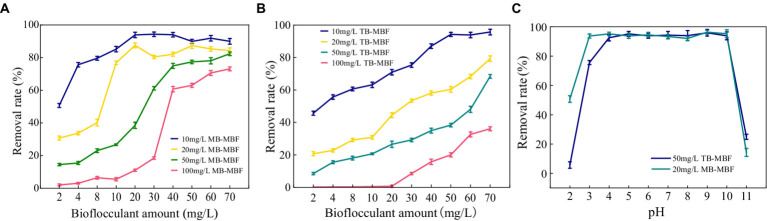
Decolorization of **(A)** methylene blue (MB) and **(B)** toluol blue (TB) by adding different amounts of MBF-35A.Effect of pH **(C)** on the decolorization of methylene blue (MB) and toluidine blue (TB).

The decolorization of methylene blue and toluol blue by MBF-35A was compared. MBF-35A exhibited maximum methylene decolorization rates of 94.31, 87.48, 82.36, and 73.07% in 10, 20, 50, and 100 mg/L dye solutions, respectively, and the corresponding MBF-35A additions were 30, 20, 70, and 70 mg, respectively (Figure 8A). In terms of toluol blue removal, MBF-35A achieved maximum decolorization rates of 95.66, 79.28, 68.36, and 36.07% in 10, 20, 50, and 100 mg/L dye solutions, respectively (Figure 8B). The pH of the reaction system is an important factor affecting the application effect of bioflocculant. The results showed that MBF-35A maintained the decolorization ability of methylene blue at pH 3–10, and the decolorization ability of p-toluidine blue was in the pH range of 4–10. At pH 2 and 11, the decolorization ability of the two dyes decreased significantly (Figure 8C).

The results imply that the environmentally friendly MBF-35A may be a promising substitute for hazardous PAM in the decolorization of dyes. It has been reported that the decolorization efficiency of BBF for methylene blue and crystal violet was 98.78 and 89.37% at a dosage of 26 g/L and 10 g/L, respectively (Mu et al., 2019). Although an excellent decolorization effect was reached, high dosages could not be ignored because of the cost.

### 3.8 Adsorption of Cr^6+^and Cu^2+^ by MBF-35A

**Figure 9:**
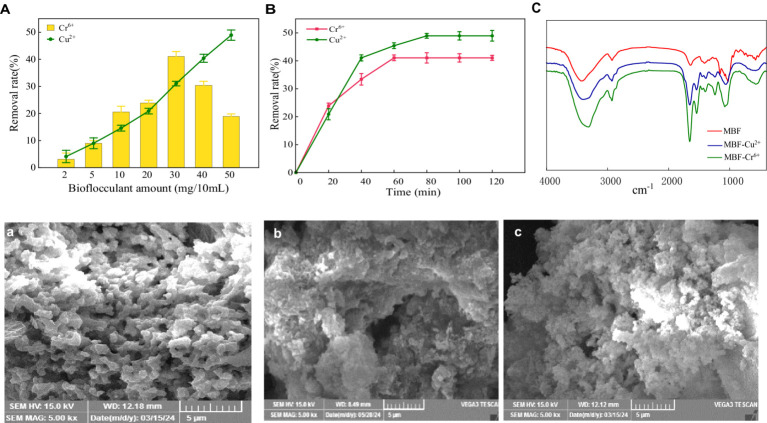
Effect of MBF-35A dosage **(A)** and reaction time **(B)** on removal of Cr^6+^ and Cu^2+^, FTIR spectra of MBF-35A after absorbing Cr^6+^ and Cu^2+^ **(C)**, SEM images of MBF-35A **(a)**, MBF-35A binding with Cr^6+^ **(b)** and MBF-35A binding with Cu^2+^ **(c)**.

Polysaccharide bio-flocculant has a large amount of negative charge, and it also has good adsorption efficiency for soluble pollutants in water, so that it can settle down the soluble pollutants better in the flocculation process, especially the positively charged heavy metal ions (Hassimi et al., 2020). In this section, the capacity and mechanism of MBF-35A in adsorbing Cr^6+^ and Cu^2+^ were explored. The results showed that the dosage of MBF-35A was a controlling parameter influencing adsorption efficiency (Figure 9A). The removal rate of Cr^6+^ by MBF-35A increased first and then decreased with the increase in MBF-35A dosage. When the dosage was 30 mg, the removal rate of Cr^6+^ reached its maximum (41.05%). Continuing to increase the input of MBF-35A will reduce the removal of Cr^6+^. The removal rate of Cu^2+^ by MBF-35A continued to increase with the increase in its dosage. When the dosage was 50 mg, the removal rate of Cu^2+^ reached its maximum, which was 48.93%. The effect of reaction time on the removal of Cr^6+^ and Cu^2+^ by MBF-35A is shown in Figure 9B. In the early stages, it showed rapid removal. With the passage of time, the removal rate slowed down, and the removal rate gradually reached equilibrium. After the addition of MBF-35A, the negatively charged group first attracted the positively charged metal ions by electrostatic force, which showed the initial rapid adsorption. As the reaction continues, the electronegative functional groups are all occupied, and the metal ions lose their binding sites. The adsorption reaches its maximum and tends to balance (Zhao et al., 2023).

The changes in functional groups before and after the adsorption of heavy metal ions by MBF-35A were further detected by infrared spectroscopy. It can be seen from this that the infrared spectrum of MBF-35A changed significantly after adsorbing Cr^6+^ and Cu^2+^ (Figure 9C). The stretching vibration peak of O-H (3430.03 cm ^−1^) shifted to 3316.21 cm ^−1^ after adsorbing Cr^6+^, and shifted to 3402.68 cm ^− 1^ after adsorbing Cu^2+^. The stretching vibration peak of C=O (1640.39 cm^−1^) shifted to 1654.55 cm^−1^ after adsorbing Cr^6+^and shifted to 1655.64 cm^−1^ after adsorbing Cu^2+^.The-C-H bending vibration peak (1412.06 cm ^−1^) shifted to 1401.00 cm ^−1^ after adsorption of Cr^6+^, and shifted to 1391.06 cm^−1^ after adsorption of Cu^2+^. The stretching vibration peak (2927.17 cm^−1^) of C-H shifts to 2929.11 cm^−1^ after adsorbing Cr^6+^ and shifted to 2930.21 cm^−1^ after adsorbing Cu^2+^. The stretching vibration peak of > P = O (1244.10 cm^−1^) shifted to 1241.09 cm^−1^ after adsorbing Cr^6+^ and shifted to 1241.16 cm^−1^ after adsorbing Cu^2+^. The stretching vibration peak (1028.37 cm^−1^) of C-O in C-O-C cyclic ether shifted to 1081.09 cm^−1^ after adsorbing Cr^6+^, and shifted to 1060.16 cm^−1^ after adsorbing Cu^2+^. The C-O-C stretching vibrations (1158.18 and 1081.06 cm^−1^) and α-glycosidic bonds disappeared after the adsorption of Cr^6+^ and Cu^2+^. The above analysis showed that various functional groups of polysaccharide components in MBF-35A were involved in the removal of Cr^6+^and Cu^2+^ (Table 2). The surface morphology of MBF-35A before and after adsorption of Cr^6+^ and Cu^2+^ was observed by scanning electron microscopy (Figures 9A–C). It can be seen that the original MBF-35A showed a smooth and porous structure, and the surface became rough and dense after adsorption of Cr^6+^ and Cu^2+^.

**Table 2:** Assignment of FTIR spectra before and after adsorbing metal ions.

Functional group	Wavenumbers (cm^−1^)		
	MBF	MBF-Cr6^+^	MBF-Cu2^+^
-OH	3430.03	3316.21	3402.68
-C-H	2927.17	2929.11	2930.21
-C=O	1640.39	1654.55	1655.64
-C-H	1412.06	1401.00	1391.06
>P=O	1244.10	1241.09	1241.16
C-O-C	1028.37	1081.09	1060.16
	1158.18	/	/
	1081.06	/	/
α-glycosidic bond	860.12	**/**	**/**

### 3.9 Treatment wastewater of meat products by MBF-35A

**Figure 10:**
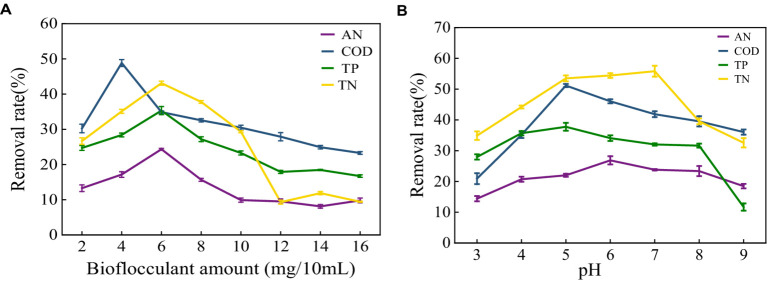
Effect of bioflocculant on meat product wastewater treatment **(A)** Removal rate of ammonia nitrogen (AN), COD, total nitrogen (TN) and total phosphorus (TP) in meat product wastewater by different bioflocculant additions; **(B)** Effect of pH on removal.

The removal effect of MBF-35A on ammonia nitrogen, COD, total phosphorus, and total nitrogen in meat product wastewater was discussed. With the increase in flocculant dosage, the removal rate increased first and then decreased (Figure 10A). At 4 mg/100 mL, the highest removal rate of COD was 48.84%. At 6 mg/100 mL, the removal effects of ammonia nitrogen, total nitrogen, and total phosphorus were the best, at 24.35, 35.33, and 43.11%, respectively. It shows that the higher the higher the dosage of bioflocculant, the better; too much or too little dosage will cause the flocculation effect to decline (Wang et al., 2023). When the flocculant dosage is small at the beginning, the flocculant is combined with the high molecular organic matter in the sewage to form flocs and settle, and the removal rate also increases. The functional groups in the structure have a strong adsorption bridging effect with the colloidal particles, which can accelerate the precipitation of colloidal particles. When the dosage of flocculant is too large, the particle aggregation in the sewage appears as a water-soluble phenomenon, and the excessive flocculant is dissolved in the sewage, which leads to an increase in organic matter concentration, an increase in electrostatic repulsion between colloids, and the phenomenon of re-stability, resulting in a decrease in the removal rate (Bai, 2020). Wang reported (Wang et al., 2023) that with the increase in flocculant dosage, the COD removal rate increased first and then decreased. When the dosage of bioflocculant was 0.3 g/L, the COD removal rate was up to 44%. The change in flocculant dosage had no significant effect on the removal rate of total nitrogen and total phosphorus. The highest removal rate of total nitrogen by bioflocculant was only 8.48%, and the removal rate of total phosphorus was between 30 and 40%. It shows that different bioflocculants have different effects on the treatment of different wastewaters.

As shown in Figure 10B, different pH conditions have a great influence on the removal of pollutants. At pH 5–7, the removal rates of ammonia nitrogen, COD, total nitrogen, and total phosphorus have the maximum values, which are 26.87, 51.16, 37.76 and 55.81%, respectively. Continuing to increase the pH value of the wastewater, the removal rates of the three have continued to decline. The pH affects the flocculation effect by acting on the surface charge of the colloidal particles and changing the properties of the flocculant. Different bioflocculants correspond to different optimum pH (Li et al., 2023). Wu studied (Wu et al., 2019) the effect of pH on the flocculation effect of bioflocculant PY-M3 when the solution pH was 4–11. The results showed that when the pH was 4–8, flocculation activity increased with the increase in pH. At pH 8, the flocculation activity reached a maximum of 92.57%, and then gradually decreased with the increase in pH.

## 4 Conclusion

A high-yield strain of bioflocculant-producing bacterium (*Bacillus subtilis* 35A) was selected. *Bacillus subtilis* 35A produced the bioflocculant (MBF-35A) most efficiently when cultivated with 10 g/L cyclodextrin and 9 g/L yeast extract as carbon and nitrogen sources, respectively. The optimal production conditions were found at 35°C for 36 h, yielding 10.47 g/L of MBF-35A. MBF-35A exhibited excellent flocculation performance with a turbidity removal efficiency of 96.57% in kaolin suspension. Adsorption and bridging are considered to be the main mechanisms of flocculation. MBF-35A was composed mainly of polysaccharides (81.74%) and proteins (16.42%), which significantly contributed to its flocculation properties. FTIR and XPS analyses revealed that MBF-35A primarily consisted of carbon, nitrogen, and oxygen, featuring functional groups (-OH, C-O, C-H, and C-O-C) conducive to flocculation. MBF-35A demonstrated over 95% decolorization efficiency for dyes and removed 41.05 and 48.93% of Cr^6+^ and Cu^2+^ ions, respectively. Application in wastewater treatment of meat products showed effective removal rates for ammonia nitrogen (26.87%), COD (51.16%), total nitrogen (37.76%), and total phosphorus (55.81%), highlighting its potential in organic waste treatment. Overall, MBF-35A not only exhibited efficient production and excellent flocculation performance as a bioflocculant but also showed significant biological and environmental benefits in dye, heavy metal ions, and organic wastewater treatment. The stability and reusability of bioflocculants need to be further studied. It may be a promising material for environmental bioremediation.

## Data Availability

The datasets presented in this study can be found in online repositories. The names of the repository/repositories and accession number(s) can be found in the article/supplementary material.
